# 570 Adult Self-Inflicted Burn Demographics in West Texas

**DOI:** 10.1093/jbcr/irae036.204

**Published:** 2024-04-17

**Authors:** Vishal Bandaru, Vivie Tran, Brandon Youssi, Coltyn Wagnon, Aya Bou Fakhreddine, Lauren Conkin, Rohan Pendse, Kaylen J Meers, John A Griswold, Alan Pang

**Affiliations:** Texas Tech University Health Sciences Center, Austin, TX; Texas Tech University Health Sciences Center School of Medicine, Lubbock, TX; Texas Tech University Health Sciences Center, Lubbock, TX; TTUHSC School of Medicine, Lubbock, TX; Texas Tech University Health Sciences Center, Austin, TX; Texas Tech University Health Sciences Center School of Medicine, Lubbock, TX; Texas Tech University Health Sciences Center, Lubbock, TX; TTUHSC School of Medicine, Lubbock, TX; Texas Tech University Health Sciences Center, Austin, TX; Texas Tech University Health Sciences Center School of Medicine, Lubbock, TX; Texas Tech University Health Sciences Center, Lubbock, TX; TTUHSC School of Medicine, Lubbock, TX; Texas Tech University Health Sciences Center, Austin, TX; Texas Tech University Health Sciences Center School of Medicine, Lubbock, TX; Texas Tech University Health Sciences Center, Lubbock, TX; TTUHSC School of Medicine, Lubbock, TX; Texas Tech University Health Sciences Center, Austin, TX; Texas Tech University Health Sciences Center School of Medicine, Lubbock, TX; Texas Tech University Health Sciences Center, Lubbock, TX; TTUHSC School of Medicine, Lubbock, TX; Texas Tech University Health Sciences Center, Austin, TX; Texas Tech University Health Sciences Center School of Medicine, Lubbock, TX; Texas Tech University Health Sciences Center, Lubbock, TX; TTUHSC School of Medicine, Lubbock, TX; Texas Tech University Health Sciences Center, Austin, TX; Texas Tech University Health Sciences Center School of Medicine, Lubbock, TX; Texas Tech University Health Sciences Center, Lubbock, TX; TTUHSC School of Medicine, Lubbock, TX; Texas Tech University Health Sciences Center, Austin, TX; Texas Tech University Health Sciences Center School of Medicine, Lubbock, TX; Texas Tech University Health Sciences Center, Lubbock, TX; TTUHSC School of Medicine, Lubbock, TX; Texas Tech University Health Sciences Center, Austin, TX; Texas Tech University Health Sciences Center School of Medicine, Lubbock, TX; Texas Tech University Health Sciences Center, Lubbock, TX; TTUHSC School of Medicine, Lubbock, TX; Texas Tech University Health Sciences Center, Austin, TX; Texas Tech University Health Sciences Center School of Medicine, Lubbock, TX; Texas Tech University Health Sciences Center, Lubbock, TX; TTUHSC School of Medicine, Lubbock, TX

## Abstract

**Introduction:**

Self-inflicted burns (SIB) are a subset of burns defined based on self-injury, sometimes also referred to as self-immolation. The United States generally reports SIB in the context of psychiatric ailment. However, due to the difference in geographic locations, the West Texas burn coverage has never been checked for SIB patients. We hypothesize that patients with SIB have higher rates of psychiatric disorders and are generally older men.

**Methods:**

We obtained a list of all patients admitted to the burn intensive care unit (BICU) with a Second/Third-degree burn. After, we verified the inclusion of patients meeting the study criteria from July 01, 2011, to July 01, 2021, ages between 18 and 89, and a completed Lund-Browder Chart. Data was collected from a mix of manual chart review from electronic health records and electronic data retrieval. Patients were split into two groups, SIB and non-intentional burns (NIB). Bivariate analysis was conducted using Mann Whitney U test and either Fisher’s exact test or Chi-square test when independent variables were categorical.

**Results:**

The initial data set (n=2,875) decreased due to missing burn documentation, burn intensive care unit admission, and incomplete records and was split into two groups: SIB (n=49) and NIB (n=1,243). The average age and percentage of male patients compared between NIB and SIB were not significant (p=0.32, p=0.39). However, TBSA, frequency of inhalation injury, burn symmetry, suicidal ideation, history of drug abuse, history of self-harm, history of mental illness, past mental health hospitalization, and mortality were all significantly higher in the SIB group (p< 0.001). There was also a statistically significant higher frequency of psychological disorders in the SIB group for depression, acute schizophrenia, borderline personality disorder, bipolar disorder (p>0.001), anxiety disorders (p=0.02), post-traumatic stress disorder (p=0.006), atypical psychosis, drug-induced psychosis, and delirium (p=0.04).

**Conclusions:**

The demographics of the West Texas SIB population are comparable to that of the United States, predominantly older male patients. Similarly, many psychiatric disorders occur at a higher frequency in SIB patients than NIB: depression, acute schizophrenia, borderline personality disorder, anxiety disorder, post—traumatic stress disorder, bipolar disorder, atypical psychosis, drug-induced psychosis, and delirium.

**Applicability of Research to Practice:**

While SIB patients make a small portion of the total burn population, there burns are often associated with worse outcomes – mortality, larger burns, and length of stay. The severity of their injuries begs the need for further analysis and hopefully prevention of SIB. Addressing psychiatric facilities and preventing these incidents from occurring may offer preventative methods.

